# Supplementary material for: miR-328-3p targets TLR2 to ameliorate oxygen-glucose deprivation injury and neutrophil extracellular trap formation in HUVECs via inhibition of the NF-κB signaling pathway
Source: PLoS One. 2024 Feb 23;19(2):e0299382. doi: 10.1371/journal.pone.0299382 (PMC10889604; doi:10.1371/journal.pone.0299382)
Supplement: S1 Raw images — (PDF) [file pone.0299382.s001.pdf]

Below are the original western blot images for TLR2, P65 NF-κB, p-P65 NF-κB, NLRP3, IL-1β, IL-18 and β-actin. The sample size is 4, and the sample order of the bands are all Normal, OGD-Normal, OGD-NC mimic, OGD-mimic, OGD-NC inhibitor, and OGD-inhibitor.

TLR2

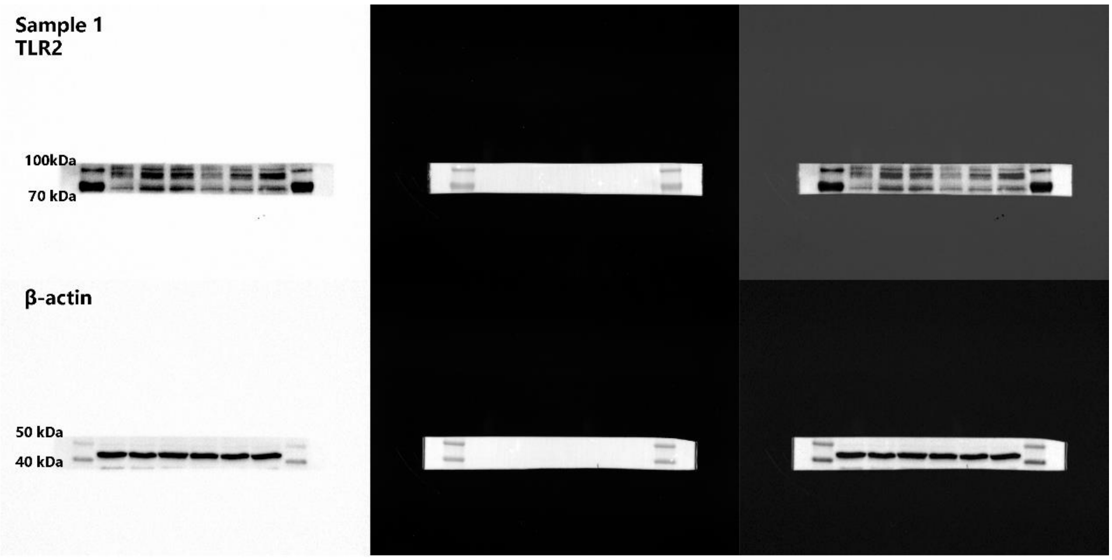

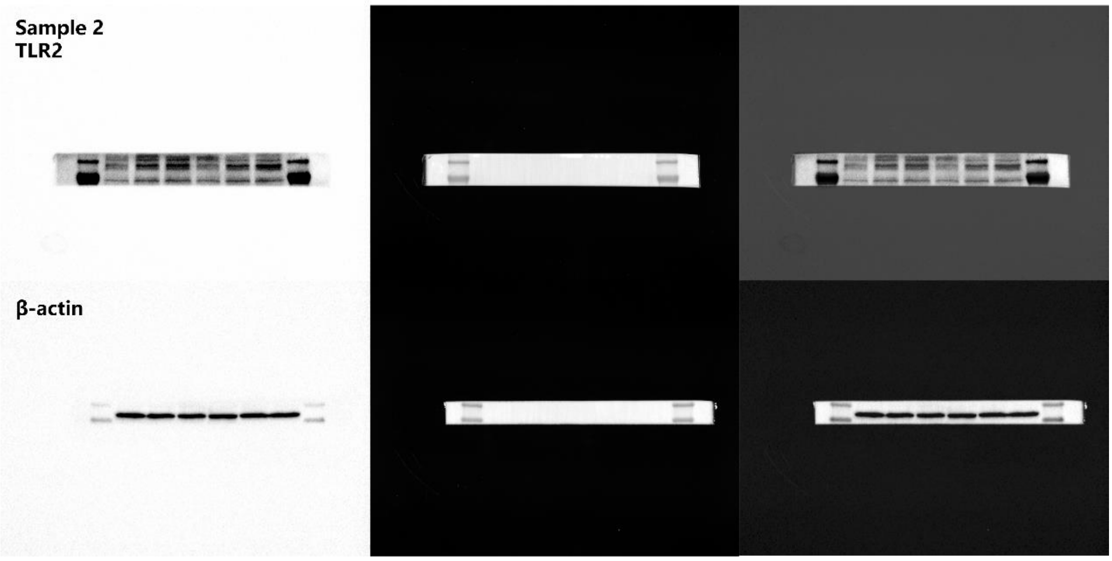

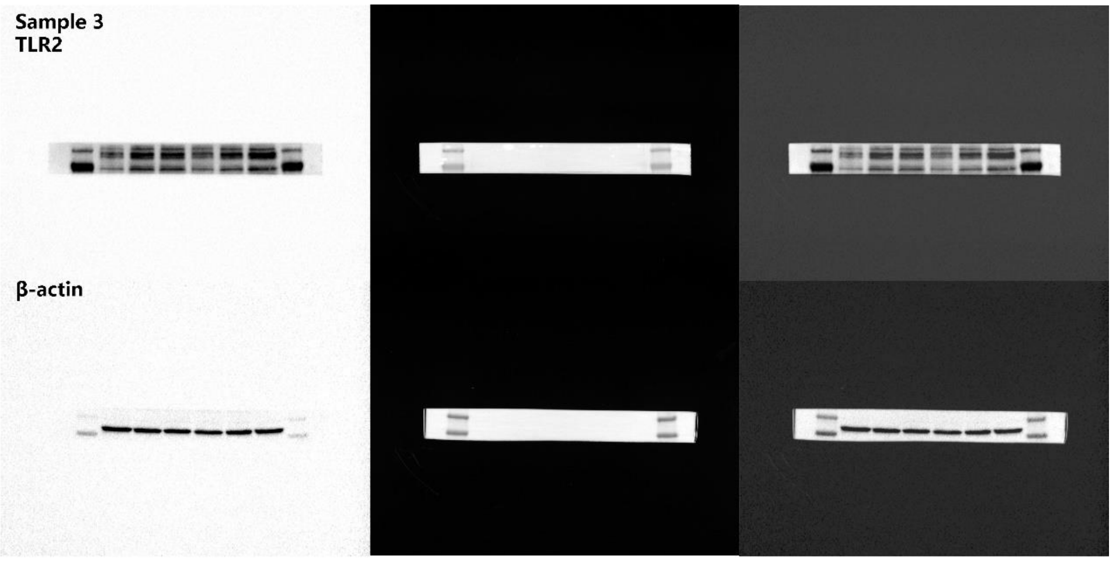
Sample 3
TLR2
β-actin

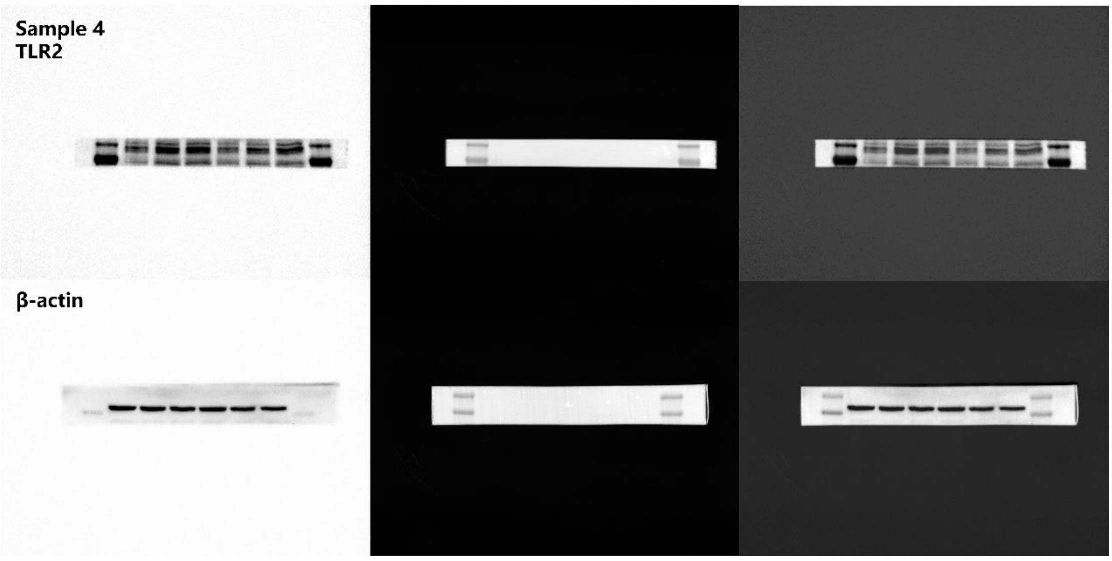
Sample 4
TLR2
β-actin

p-P65 NF-κB and P65 NF-κB

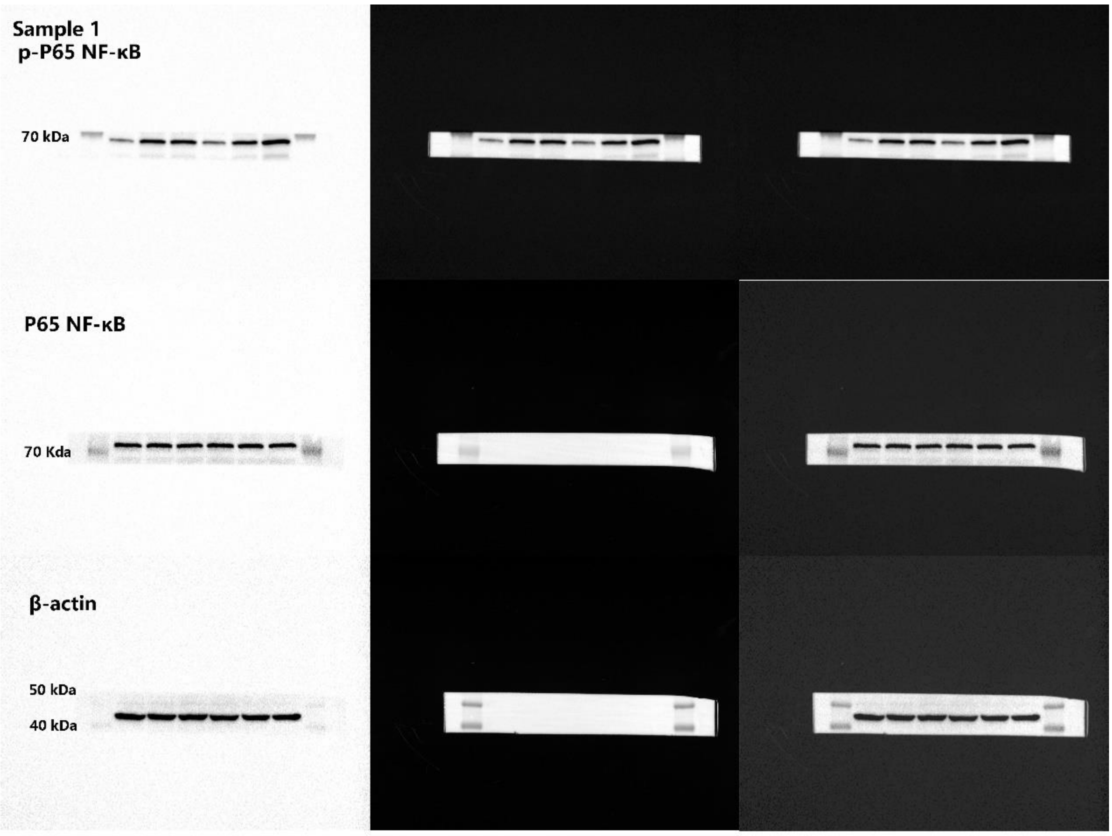

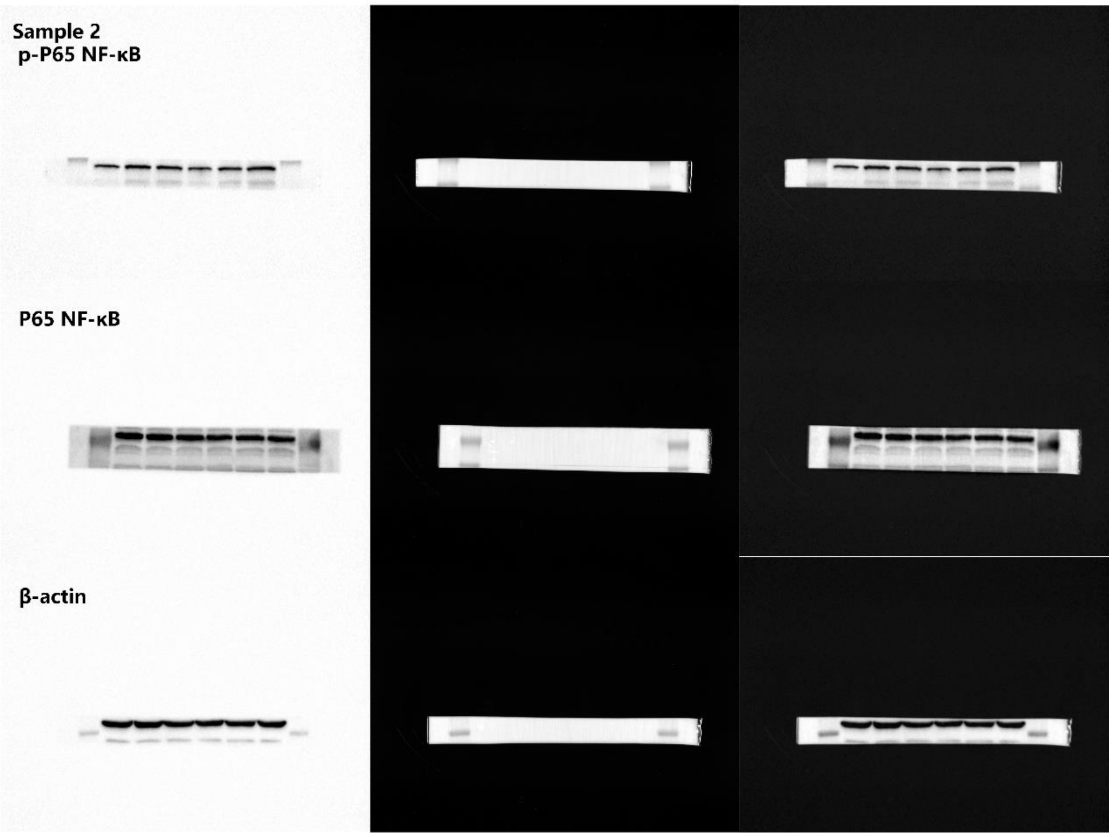

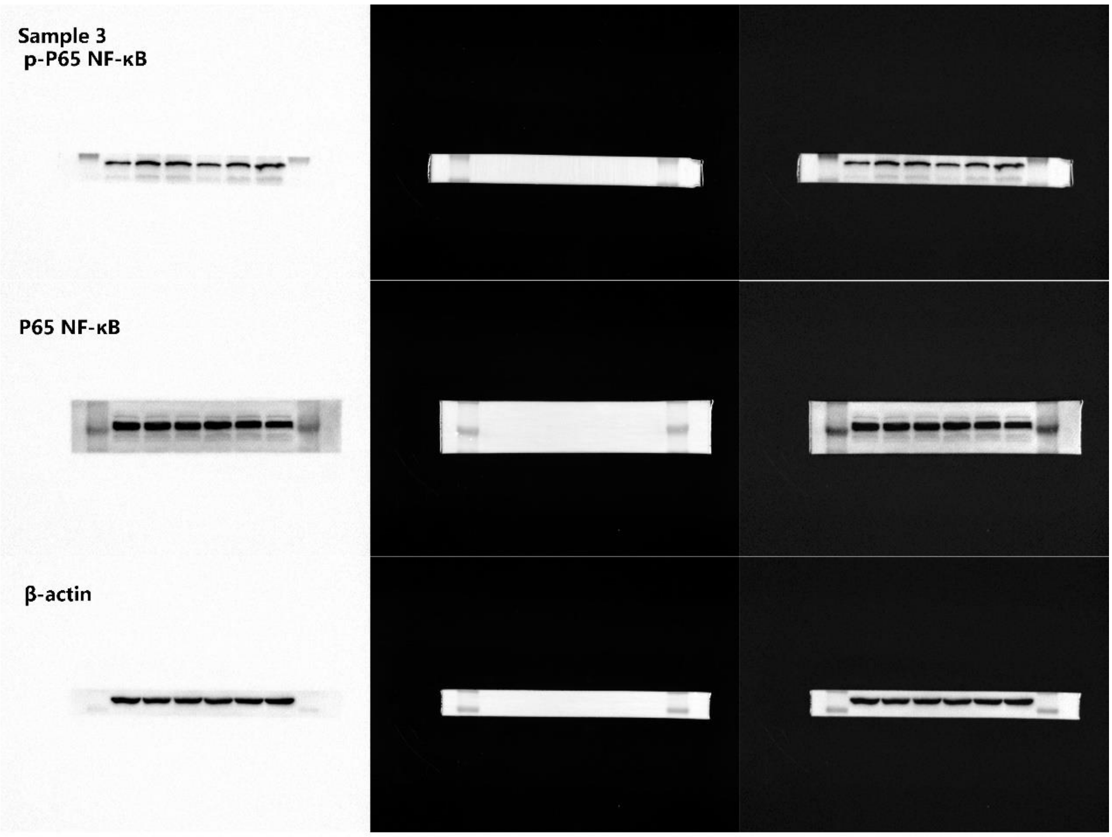
Sample 3
p-P65 NF-κB
P65 NF-κB
β-actin

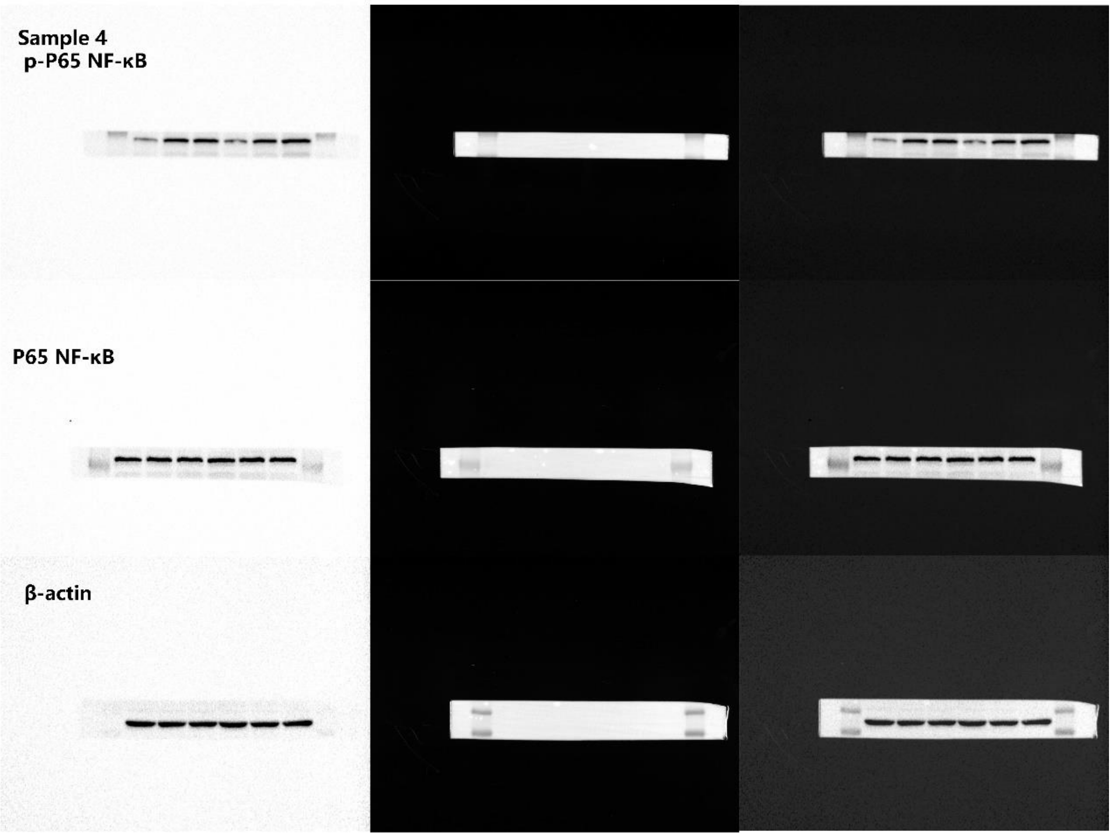
Sample 4
p-P65 NF-κB
P65 NF-κB
β-actin

NLRP3

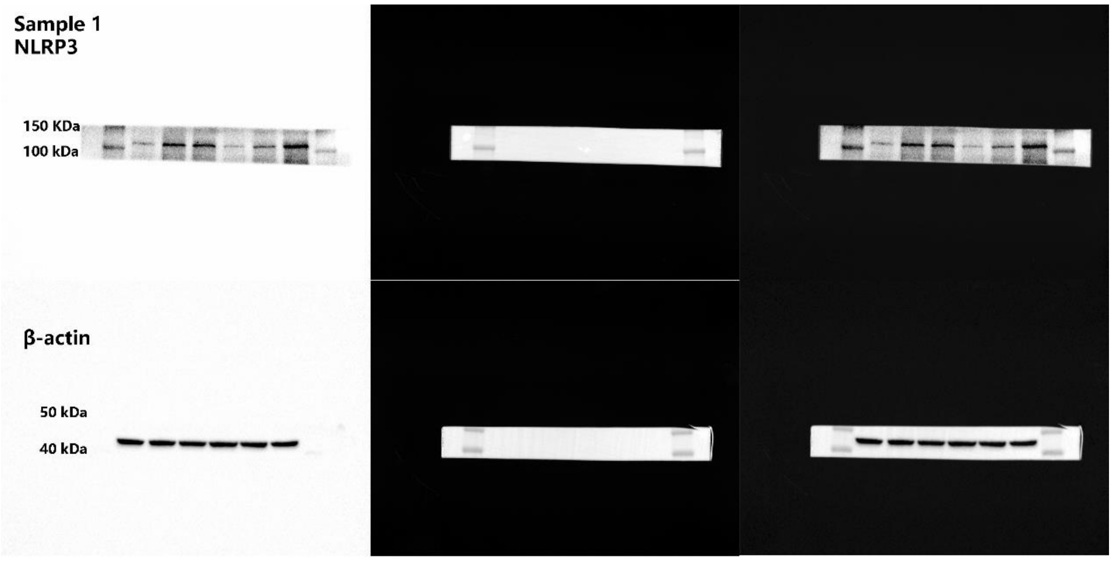
Sample 1
NLRP3
150 KDa
100 kDa
β-actin
50 kDa
40 kDa

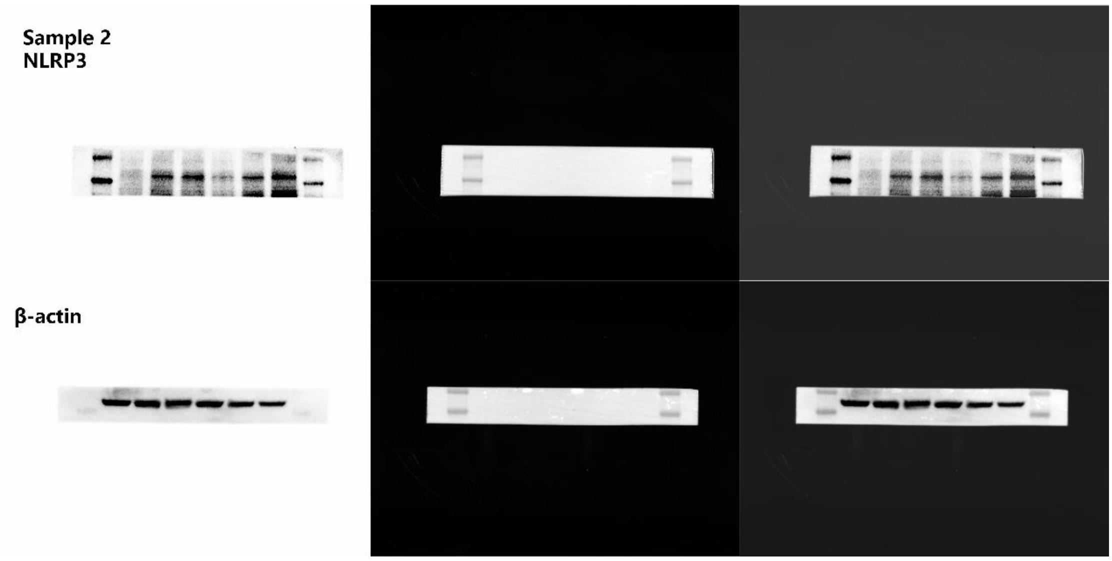
Sample 2
NLRP3
β-actin

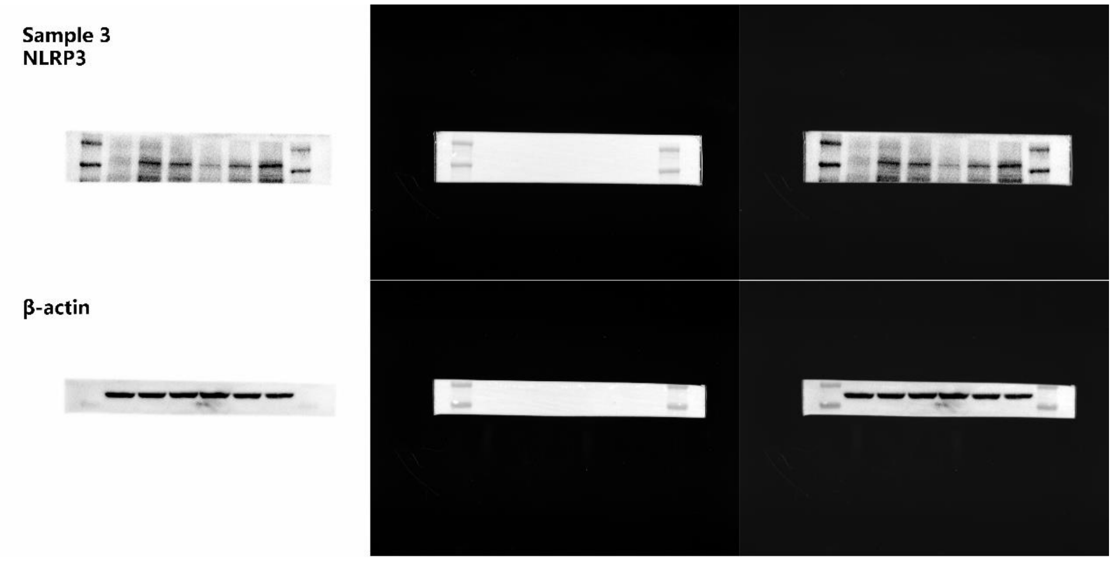
Sample 3
NLRP3
β-actin

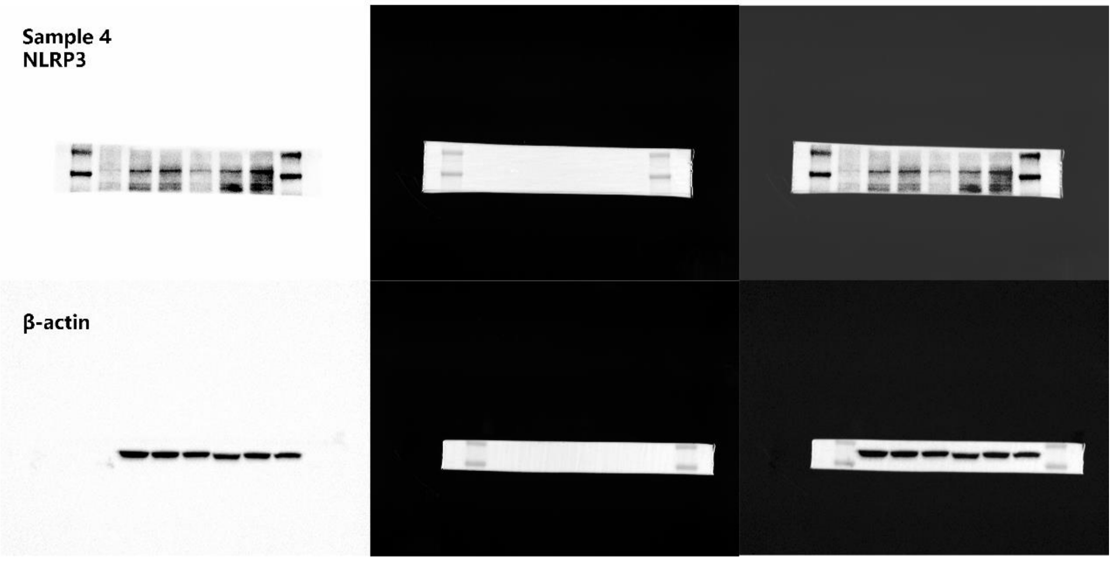
Sample 4
NLRP3
β-actin

IL-1β

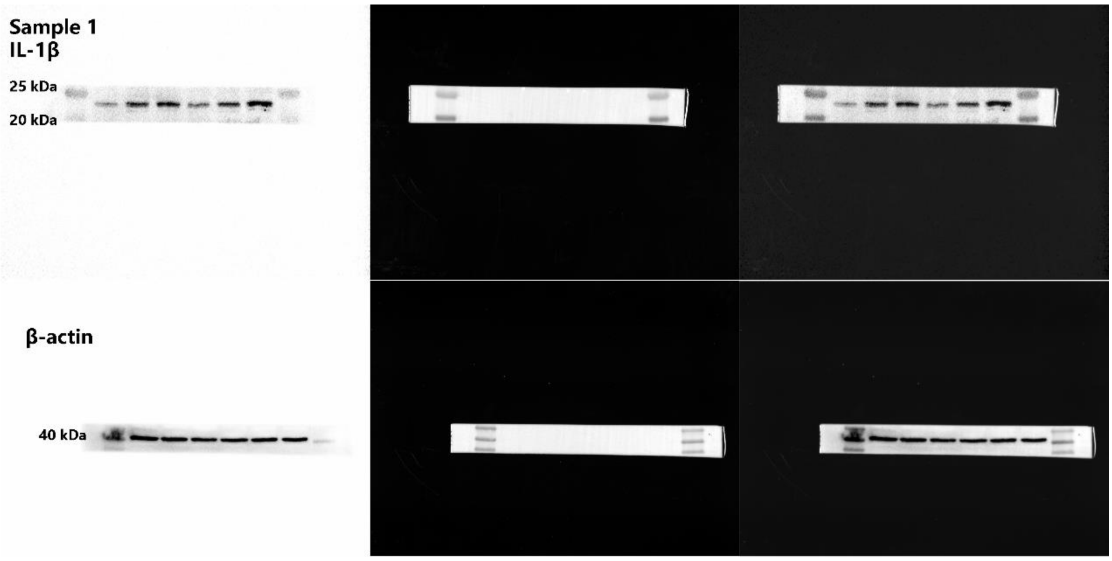

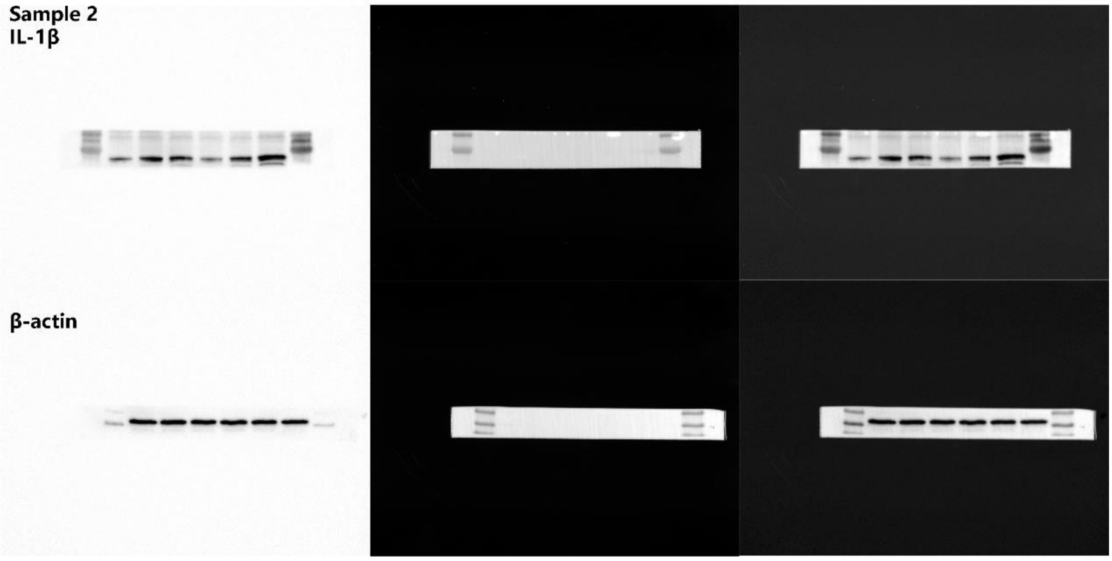

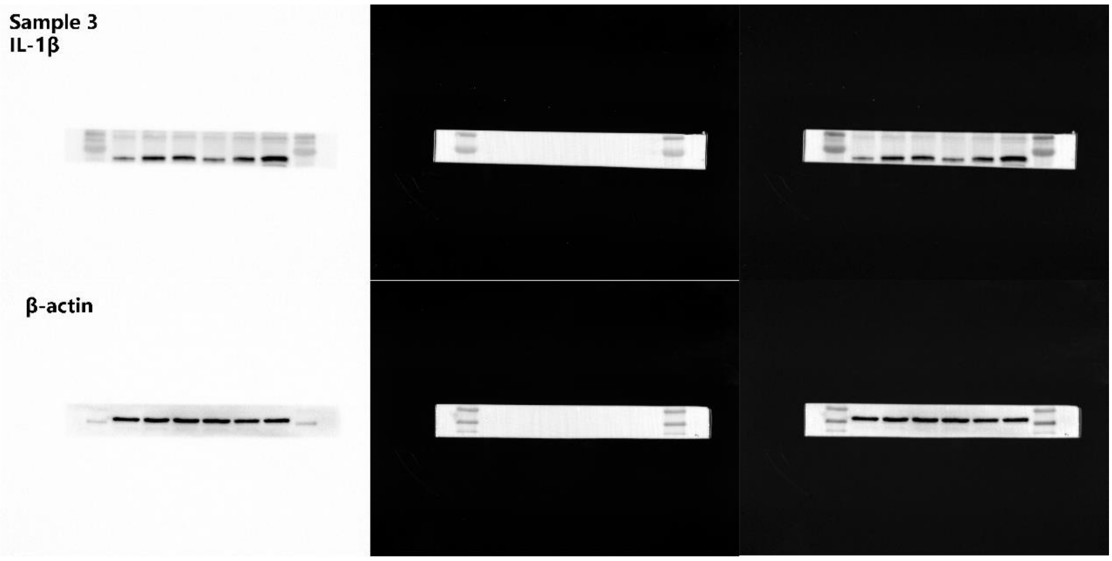
Sample 3
IL-1β
β-actin

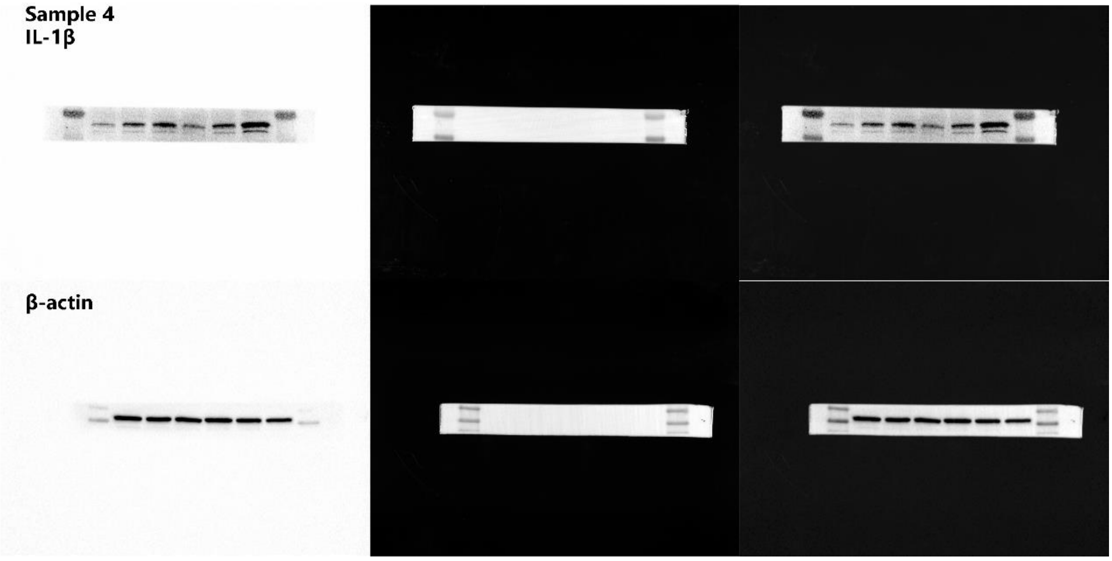
Sample 4
IL-1β
β-actin

IL-18

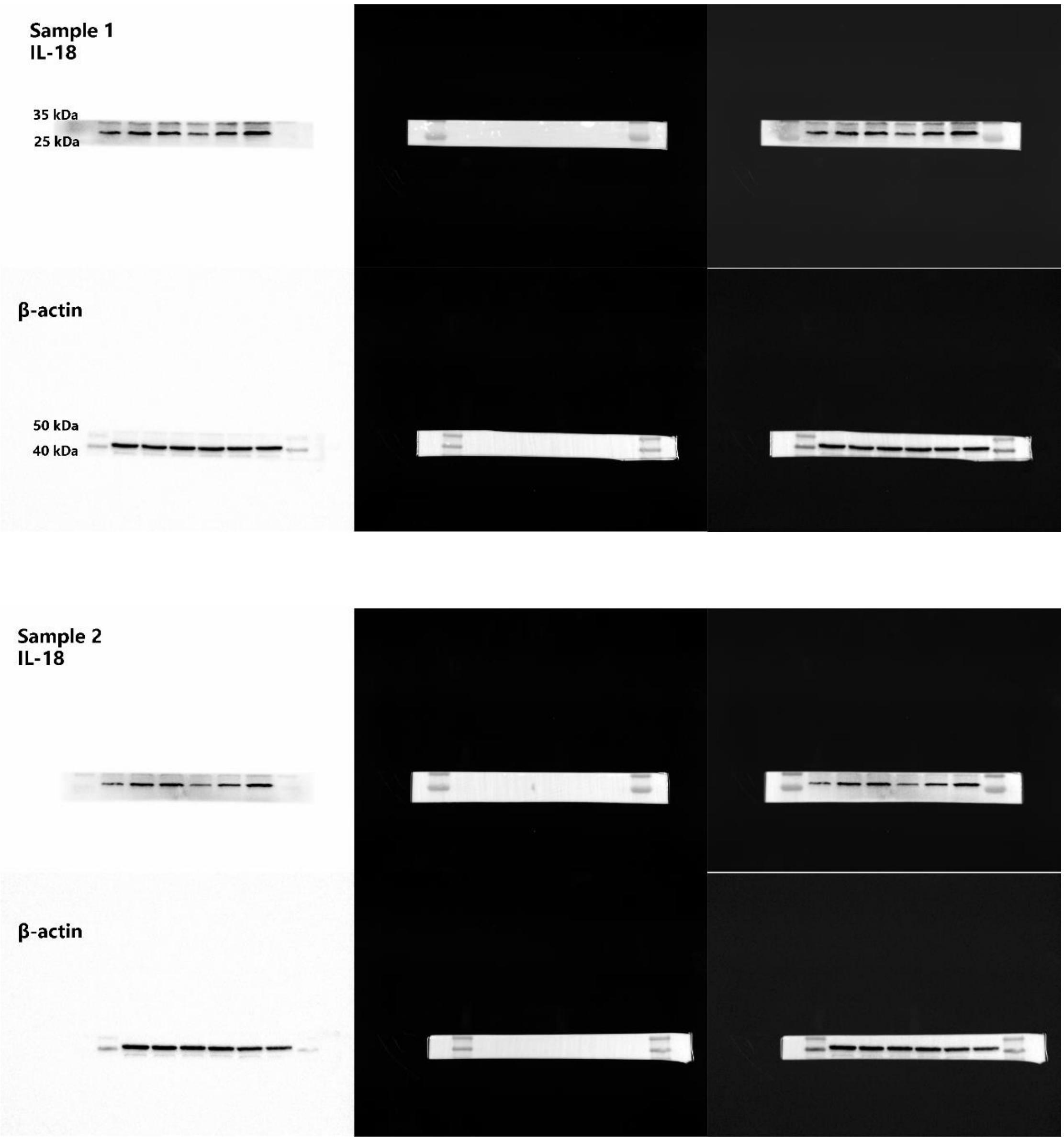
Sample 1
IL-18
35 kDa
25 kDa
β-actin
50 kDa
40 kDa
Sample 2
IL-18
β-actin

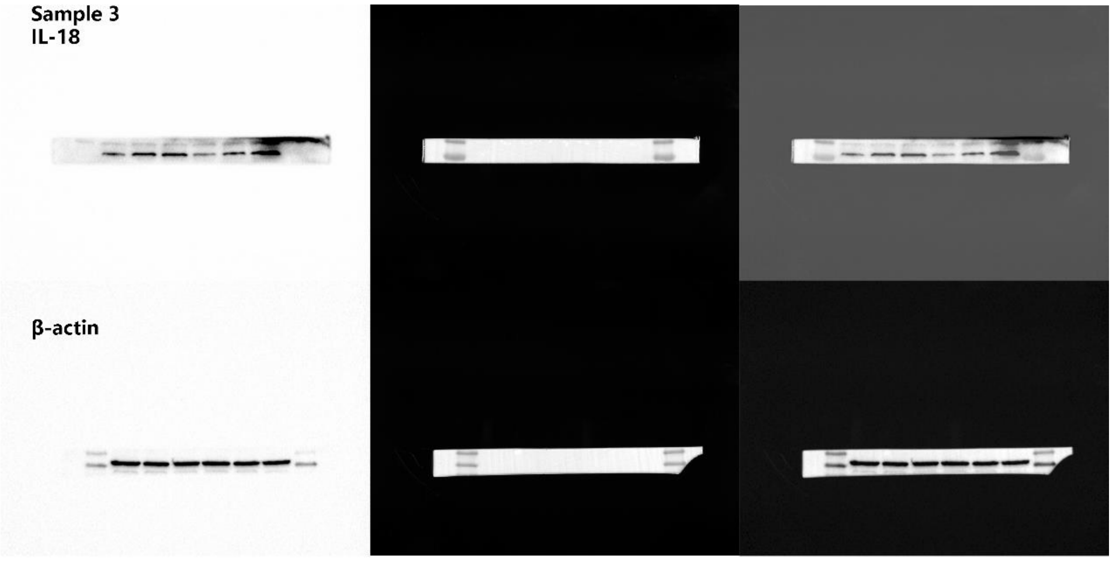
Sample 3
IL-18
β-actin

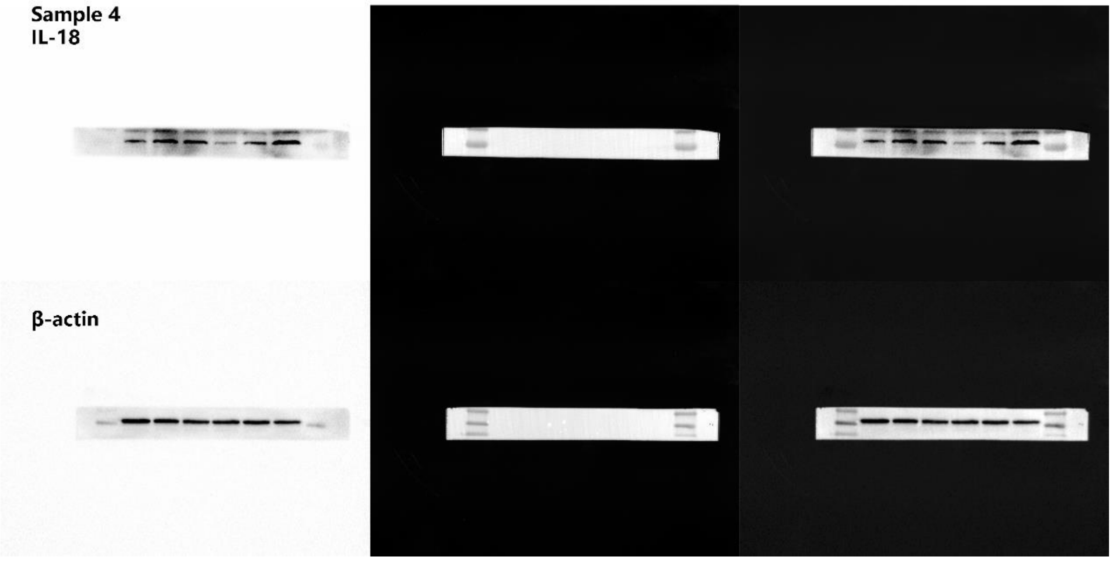
Sample 4
IL-18
β-actin
